# An *in-vitro* transcription assay for development of Rotavirus VP7

**Published:** 2017-06

**Authors:** Shahram Jalilian, Ali Teimoori, Manoochehr Makvandi, Milad Zandi

**Affiliations:** 1 Infectious and Tropical Diseases Research Center, Health Research Institute, Ahvaz Jundishapur University of Medical Sciences, Ahvaz, Iran; 2 Department of Virology, School of Medicine, Ahvaz Jundishapur University of Medical Sciences, Ahvaz, Iran

**Keywords:** *In vitro* transcription, Rotavirus, Restriction enzyme, T7 RNA polymerase, VP7 segment

## Abstract

**Background and Objectives:**

Human rotavirus (RV) is responsible for most cases of acute gastroenteritis in infants, worldwide. Today, *in vitro* transcription (IVT) assay is widely used to develop efficient RNA for the biological experiments such as gene function analysis and reverse genetics. The aim of this study was to develop optimal full-length transcripts of the VP7 segment, using *in vitro* transcription assay.

**Materials and Methods:**

Special primers were designed in order to synthesize VP7 sequence of sense RNA in the process of IVT using T7 RNA polymerase. RT-PCR was performed using forward and reverse primers, containing T7 promoter sequence and *BstUI* restriction enzyme site, respectively. In order to synthesize ssRNA VP7, in accordance with the IVT technique, RV4-VP7 fragment was subcloned into PTZ57 R/T plasmid and digested by *BstUI* enzyme.

**Results:**

The sequencing of the VP7 gene showed 99% identity withVP7 gene of rotavirus RV4 strain (Sequence ID: M64666.1). The analysis of purity of DNA fragment and ssRNA VP7 segment revealed that OD ratio of A260/A280 and quantity of nucleic acids were (1.9, 0.036 μg/μL) and (2.02, 0.98 μg/μL), respectively.

**Conclusion:**

In the present study, a modified methodology of RNA synthetase was described by IVT assay, using T7RNA polymerase in order to transcribe the full-length transcripts of human VP7-RV4 strain. This method is applicable for reverse genetic approaches, especially for the production of reassortant RV vaccine.

## INTRODUCTION

*In vitro* transcription (IVT) is a simple procedure that facilitates the synthesis of RNA molecules from less than 0.3 to 5 kb of DNA (1). Studies on IVT will open up new avenues for RNA researchers to synthesize RNAs by using T7 RNA polymerase (RNAP). It also enables the synthesis of *in vitro* full-length viral RNA with high efficiency when compared with normal cell transcription (2). This technique could be useful for examination of antigenic diversity, characterization of reassortant, and applicable in more sophisticated techniques named reverse genetics, which has dramatically remodeled the investigation of RNA virus (3). In 2006, the first reverse genetic model was developed for Rotavirus (RV) reassortant (4). RVs are members of the *Reoviridae* family and comprise of 11 segments of double-stranded RNA. RVs are the main causal agent of acute gastroenteritis in infants and young children, especially in developing countries. Based on the diversity of VP6 gene, RVs have been classified into seven groups (A–G). Group A (G1P8) is implicated in acute gastroenteritis in many regions, including Iran (5). The World Health Organization (WHO) estimated that RV mortality rate in children below five years was at 215,000 (range: 197,000–233,000) per year worldwide (6). Owing to the lack of specific treatment, vaccination is the only preventive measure against infection in young children. Till date, the reassortant-developed RV vaccines were based on insertion of a human RV segment in bovine RV strain, where in this phenomenon occurs as a natural selection (7). Today, researchers have made efforts to develop a new RV vaccine based on reverse genetics technique. Two major outer capsid proteins of rotavirus, VP7 and VP4, contain neutralization antigens. Both VP4 (P-type) and VP7 (G-type) are protective and neutralizing antibodies; and glycosylated VP7 is a major component of outer capsid proteins of RVs (8).

For this purpose, the present study was carried out in order to establish a new pattern to synthesize VP7 ssRNA-RV4 strain, which ultimately led to the synthesis of VP7 dsRNA *in vivo*. The outcome of this event is promising. This approach can be used as a potential new tool for RV vaccine development, in the near future.

## MATERIALS AND METHODS

### Virus cultivation.

MA104 cell line derived from Rhesus monkey kidney (routinely maintained in our laboratory) cultured in a 75-cm^2^ flask (approximately 7.5 ×10^6^ cells) and rinsed three times with 10 ml sterile phosphate-buffered saline (PBS). Pre-activated human rotavirus G1P8 strain RV4 (MOI 0.1) (purchased from European Collection of Authenticated Cell Cultures [ECACC]) with10 μg/ml L-1-tosylamido-2-phenylethyl chloromethyl ketone (TPCK) trypsin (Thermo Fisher Scientific, USA) was inoculated with cell culture and incubated at 37°C for one hour. Meanwhile, in order to preclude cells from drying, the flask was stirred at 10-minute intervals. Then 15 ml of Dulbecco’s Modified Eagle’s medium (DMEM) plus trypsin at a final concentration of <2 μg/ml was added to tissue cells. The flask was incubated at 37°C, 5% CO_2_ for three days till the complete cytopathic effect (CPE) was observed. One freeze and thaw cycle was performed, then the infected cells were transferred into 50 ml conical tube. Centrifugation (8000 rpm for 10min) was carried out in order to remove all cellular debris. The clarified viruses were aliquoted in 1.5 ml microtubes and stored at −80°C, until further use.

### Rotavirus titration.

One of the approaches to determine the quantity of RV infection is the plaque assay. Initially MA104 cells were distributed into six-well plates with an equivalent density of 3.0 × 10^5^ cells per well, and incubated at 37°C, in 5% CO_2_ humidified incubator for 2 to 3 days. Once the cells were quite confluent in mono-layers, the medium was removed and MA104 cells were washed with PBS. Serial dilutions of 10^−4^ to 10^−6^ of pre-activated RV were prepared. Subsequently, 400μl of aliquots were inoculated onto each six well plates with confluent mono-layer cells. Plates were incubated at 37°C for 1hr. Then they were allowed to adsorb for 1 hr with rotational agitation every 10 minutes. The inoculums were removed and the agarose overlay comprised of 1% agarose and 2x DMEM with 2 μg/ml trypsin were applied at 45°C. The plates were incubated 72 hr at 37°C for plaque formation. In order to visualize better the plaque formation, the mixture of 1:1 of 2XDMEM containing 50 μg/ml neutral red and 1% agarose overlay was subjected to the first layer. Once the agarose had solidified, 0.02 mg/ml neutral red was added to each well. Then plates were incubated at 37°C for 24 hr.

### Isolation and purification of human rotavirus from unique plaques.

Each single plaque present in each well was picked up by sterile pipette Pasteur and put into a separate 1.5 ml micro-tube containing 650 μl DMEM. Tubes were kept at 4°C overnight, to allow the virus to be eluted from agarose into DMEM solution. In order to activate RV, 10 μg/ml trypsin was added into each tube. Tubes were kept at 37°C for 1 hr. Mono-layers of MA104 cells was seeded in 24-well plates. Then the microtubes contents were inoculated onto each well with the confluent monolayer. Plates were incubated at 37°C until cytopathic effect (CPE) was observed. Two freeze and thaw cycles was carried out to recover the complete contents of each well.

### Total RNA extraction.

The total RNA was extracted from each single plaque using RNX-PLUS (SinaClon, Iran), in accordance with manufacturer’s instructions. An additional step of denaturation was performed at 55°C to 65°C for 10 minutes to relax RNA, which could be used for downstream applications, such as reverse transcription-polymerase chain reaction (RT-PCR) and polyacrylamide gel electrophoresis (PAGE).

### SDS-PAGE and silver nitrate staining.

SDS-PAGE as the best method for visualizing dsRNA was used to evaluate the extracted dsRNA RV with silver stains.

The extracted RV RNA from a single plaque was subjected to electrophoresis on 12% sodium dodecyl sulphate-polyacrylamide gels (SDS-PAGE). The gel electrophoresis was run at 25 mAmp for 14 hr. For fixation and staining, the gel was washed with distilled water and placed in a solution containing 5% ethanol, 0.1% AgNO_3_ and 1% nitric acid for five minutes. Again, the gel was rinsed one time with distilled water for 10 seconds, then the solution containing 0.65% Na_2_CO_3_, 0.4% HCOH, and 1.3% NAOH was poured onto the gel, and shaken slowly in order to visualize the segments of rotavirus. The stopping solution containing 5% ethanol and 1% nitric acid was added after 1 minute, and the gel was rinsed with distilled water.

### Isolation and verification of VP7 segment of human Rotavirus RV4 strain.

The SDS-PAGE was performed to isolate the VP7 segment of RV4-Rotavirus. Following Ethidium bromide staining (0.5μg/ ml EtBr in water) on the polyacrylamide gel, two near fragments (7, 8 and 9), was cut with a sterile scalpel and each piece of the gel placed in one separate tube containing DEPC-water. The tubes were stored at 4°C for 24 hours. Finally, the RNA extraction was carried out with RNX-PLUS kit and RT-PCR was performed with G-con (1044–1062 nt) and G1 (aBT1) (314–335 nt) primers (Table 1).

**Table 1.** The list and sequence of primers used for RT-PCR reactions, in the process of verification of VP7 segment and IVT assay

**Primers for Verification of VP7 segment**	
G-con	GGTCACATCATACAATTCT
G1	CAAGTACTCAAATCAATGATGG
**Primers for IVT**	
Forward primer	TAATACGACTCACTATAGGCTTTAAAAGAGAGAATTCCC
reverse primer	ATCGCGGTCACATCGAACAATTCTAATCT

### Primer design for IVT.

The primers were designed using Gene runner 5.1.0.5 Beta software. Forward and reverse primers consist of T7 RAN promoter sequence and *BstUI* restriction enzyme site, respectively (Table 1). The specificity of the primers was checked and verified with the primer blast (https://www.ncbi.nlm.nih.gov/tools/primer-blast).

### Reverse transcription and amplification of DNA.

In order to enhance the efficiency of the cDNA synthesis and to separate dsRNA, dsRNA of RV was heated for 5 minutes at 97°C. The synthesis of RNA from cDNA was performed using cDNA synthesis kit (Qiagen, Inc., Valencia, CA) in accordance with the manufacturer’s instructions. The specific forward and reverse primers were designed for the entire region of segment 9 (VP7), and amplified using the following program: initial denaturation at 95°C for 5 minutes, followed by 30 cycles of 30 s at 95°C, 30 s at 60°C, and 60s at 72°C, and a final extension at 72°C for 10 min (to facilitate the 3′-adenine addition). The product was analysed on a 1% agarose gel. PCR product was purified (Nucleic Acid Purification Kit, favorgen, Taiwan). The sequencing of the PCR product was performed and confirmed by ABI 3730XL DNA Analyser (Bioneer, Co, Korean). Analysis of sequencing was carried out by the nucleotide blast (https://blast.ncbi.nlm.nih.gov/Blast.cgi) and sequence alignment (MEGA6 program).

### TA cloning.

The purified PCR product with 1:3 ratio of 100 ng of 2886 bp linearized vector PTZ57 R/T (Fermentas, Lithuania) and 112.7 ng of 1085 bp PCR product was subcloned into vector. Ligation was performed with 1μl T4 DNA ligase (5u/μl Invitrogen, Carlsbad, CA, USA), 4 μl ligation buffer 5X and water nuclease-free into total volume of 20 μl. The mixture incubated at room temperature for 1hr followed by incubation at 4°C overnight.

The fresh *E. coli* DH5α (TaKaRa Biotechnology Co, Dalian, China) was prepared, precipitated, and then cold CaCl_2_ transformation was carried out by adding 2 μl ligated plasmid to 50 μl of *E. coli* DH5 α. The transformed *E. coli* streaked out on LB (Luria-Bertani) agar plates (50mg/ml of Ampicillin) with 80μl of 100 mg/ml IPTG (Isopropyl β-D-1-thiogalactopyranoside) and 40 μl of 20 mg/ml X-gal (5-Bromo-4-chloro-3-indolyl-β-D-galactoside). After incubation at 37°C for 18hr, the white colonies were selected. Each selected colony was inoculated into a tube containing 5 ml LB broth. Tubes were kept overnight in a shaker incubator at 37°C.

In the next step, the plasmid was extracted, using (plasmid DNA Extraction Mini Kit). The extracted plasmid was digested with the *Bstu-1* restriction enzyme (New England Biolabs, United States) at 60°C for 2 hrs. In order to confirm the digestion process, the product was run on a 1% agarose gel and visualized under UV trans-illuminator. After cleaning up the digested VP7 segments, the quantification was done by NanoDrop One^c^ (Thermo scientific, USA).

### *In vitro* transcription assay.

VP7 segment’s clean-up contained T7 RNAP promoter site upstream of the VP7 sequence and was applied as a template for *in vitro* transcription using mMESSAGE mMACHINE® Kits AM1344 (Life Technologies, Thermo Fisher Scientific Inc.).

When sense VP7 RNA (the same sequence as the mRNA) is required, it is important using the appropriate phage promoter site by placing on the 5′-end of the VP7 sequence. On the other hand, the final DNA template essentially participates in the reconstruction process of VP7 RNA sequence during *in vitro* transcription, having a right T7 RNAP promoter site upstream of the sequence to be transcribed.

A mixture containing 2μl of 10X reaction buffer, 10 μl 2X NTP, 2 μl T7 RNAP enzyme, 0.2 μg template and nuclease-free water up to final volume of 20 μl was prepared. The mixture was incubated at 37°C for two hours, then 2 μl TURBO DNase was added in order to eliminate the DNA template and again incubation was performed for 30 min at 37°C.

In order to obtain high purity RNA, proteins, enzymes, and un-incorporated nucleotides were removed and precipitated by lithium chloride (0.5M to 1.0M LiCl). Given that, the supernatant containing RNAs greater than 0.1 μg/μl or larger than 300 bp will be precipitated with LiCl. The precipitation of RNA was carried out by adding 30 μl of LiCl solution, mixed, incubated at −20°C for 30 minutes and centrifuged at 14000 RCF in 4°C for 15 minutes. The supernatant was removed carefully and the pellet rinsed with 1 ml 70% cold ethanol. High-speed centrifugation was performed in order to remove additional nucleotides. The 70% ethanol was evaporated and the RNA resuspended in 20 μl nuclease-free water.

### The quantity and quality RNA synthesized.

The concentration of VP7 segment was measured with NanoDrop One^c^ and stored at –70°C, until further use. In order to confirm the RNA yield, RT-PCR was performed with cDNA and RNA using minus RT as a control. Finally, the quality of purified synthesized RNA (1 μg) was checked by electrophoresis on 1% agarose gel.

## RESULTS

**Fig. 1:**
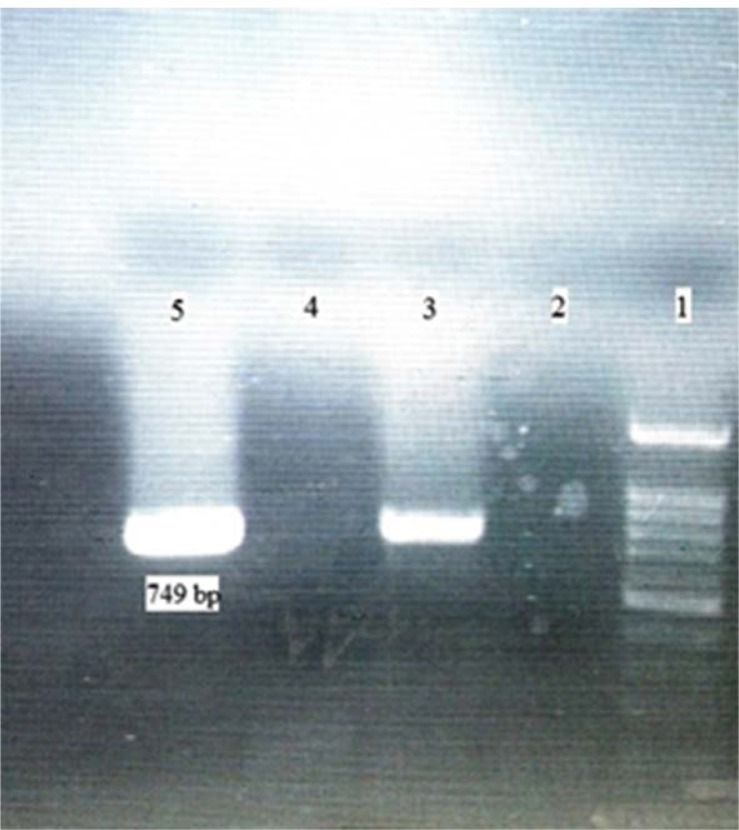
The result of PCR amplification for verification of VP7 segment of human Rotavirus RV4 strain (G1P8), lane (1) 100 bp DNA ladder (CinnaClon), lane (2) negative control, lane (3) PCR positive control (749 bp), lane (4) negative PCR result for segment 7& 8, lane (5) positive PCR result for segment 9 (749bp).

The titre of RV4 determined by plaque assay was 5×10^5^ particles per microlitre. The purified RV4 strain showed 11 dsRNA segments by SDS-PAGE and silver staining. Generally, the segments’ electrophoresis patterns of the majority of human rotavirus are as follows: 1-VP1, 2-VP2, 3-VP3, 4-VP4, 5-NSP1, 6-VP6, 7-NSP3, 8-NSP2, 9-VP7, 10-NSP4, and 11-NSP5. Meanwhile, segments 2 and 3, as well as 7 and 8, ran very close together in term of size. The VP7 segment moves a small distance from the (7, 8) segments (Fig. 1).

The sequencing of extracted plasmid, using M13 universal primers revealed that the inserted DNA has over 99% homology with human rotavirus RV4 strain major outer capsid glycoprotein (VP7) gene (Sequence ID: M64666.1).

**Fig. 2:**
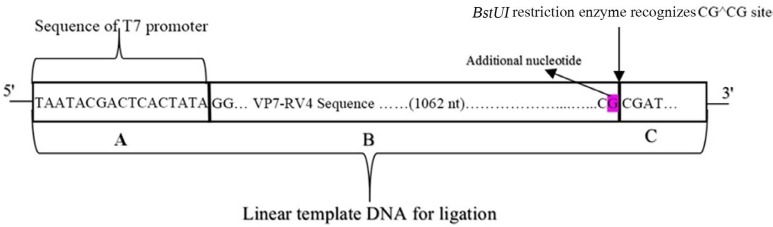
Schematic view of linear template DNA of VP7 used for ligation: A: Sequence of T7promoter located upstream of 5′-terminus. B: Sequence of the VP7 gene, belong to segment 9 of human rotavirus strain RV4. Additional G nucleotide at the 3’ end of VP7 Sequence is the outcome of cleavage with RE-*Bstu-1*. C: Part of the vector, removed after blunt end cutting with restriction enzyme.

The linear template DNA, which was amplified by PCR and subcloned into the PTZ57 R/T vector, composed of Rotavirus VP7 sequence (RV4 strain) plus T7 promoter and Bstu-1 RE-site, upstream and downstream, respectively (Fig. 2).

**Fig. 3:**
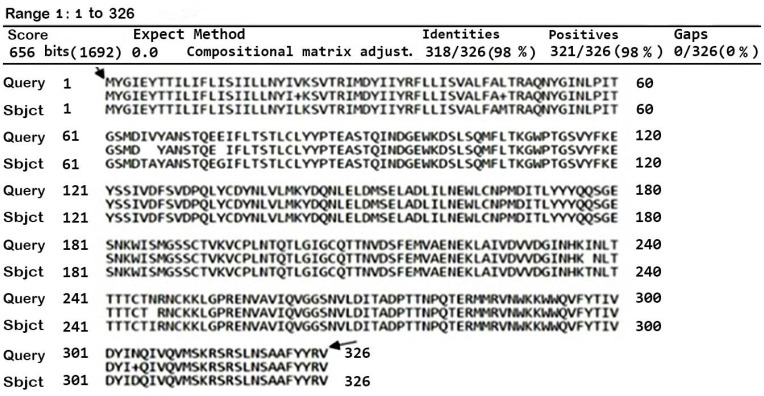
VP7 Blast protein analysis (designed with GeneRunner program) against database finding similarity with sequence ID: M64666.1.

The quantity of DNA fragment obtained by *BstUI* RE (0.036 μg/μL) and concentration of ssRNA VP7 segment, acquired from the IVT assay (0.98 μg/μL), was carried out by NanoDrop One^c^. The ratio of absorbance at 260 nm and 280 nm (A260/A280) is utilized to evaluate the RNA and DNA purity. A ratio of ∼2.0 and ∼1.8 are usually recognized as ‘pure’ for RNA and DNA, respectively. In our experiment, the target DNA result of enzymatic digestion (1.9) and RNA synthesized (2.02) delivered an acceptable purity at A260/A280 ratio. According to Fig. 3, the PTZ 57 R/T-VP7 clone sequencing revealed 10 point mutations that lead to eight amino acid (aa) substitute as compared to the reference VP7-RV4 sequence (GeneBank Sequence ID: M64666.1). Protein blast indicated approximately that each of this aa change also exists in other VP7 human RV group A.

## DISCUSSION

Today, most of the studies on reassortant vaccines are focused on VP7, as it induces production of neutralizing antibodies (9). Based on the electrophoretic mobility pattern, the polyacrylamide gel electrophoresis is a gold standard test for determination of 11 segments of RV genes (dsRNAs) (10). Several studies have shown that the position of 7-8-9 segments in the human rotavirus (group A) strains are very close to each other (11, 12). Since no study has illustrated VP7 segment isolation and no data exist regarding its exact location in a G1P8 strain of human Rotavirus RV4, the polyacrylamide gel segment was cut and subsequently amplified with RT-PCR. Results of Fig. 1, show the segment 9 of Rotavirus-RV4 is VP7.

Rotavirus strain G1P8 is the most common RV infection which has been reported in Iran (13). Since there is no specific treatment, the vaccination against RV is the only approach to prevent the infection. The current investigations exhibited that the VP7 segment was found to be one of the best candidates for reassortant vaccines (7–9). Thus we have used a VP7 segment of RV4-strain (G1p8) for *in vitro* transcription assay, in order to produce Rotavirus VP7.

In order to apply reverse genetic for RV segments, several methods have been described. In the most prominent works, the T7 RNAP was used by BSR5/T7 cells or recombinant vaccinia virus system (14, 15). In contrast, we have used *in vitro* transcription technique using T7 RNAP, which was included in the kitmMESSAGE mMACHINE® AM1344 (1). To do this, T7 promoter sequence class III (TAATACGACTCACTATAGGG) was placed upstream of the flanking 5′-end of the forward primer.

T7 promoters have G’s at +1, to +3 end sequence, and the first two Gs are critical for the transcriptional process. As regards, the +1 base is the first to participate in RNA transcription (16). On the other hand, the 5′ end of the sequence of human rotavirus RV4 starts with two G residues. Eventually, the guanine residues were omitted from the sequence of the T7 promoter (Forward primer:

$$\begin{array}{l} \text{Sequence}\;\text{of}\;\text{T}7\;\text{promoter}\\ 5\prime -\underline{\text{TAATACGACTCACTATA}}\overset{+1+2}{\text{GG}}\text{CTTTAAAA}\\ \;\;\;\text{GAGAGAATTCCC}-3\prime \end{array}$$

with retained structural VP7 RV4 sequence.

In order to synthesize functional RNA, the correct sequence is required for molecular analysis, such as X-ray, NMR and ligation reactions (17). DNA (Taq polymerase) and T7 RNAP usually cause premature termination by falling off the DNA template before finishing their job. These irregular forms of amplicons cannot be used for identifying and isolating the correct full-length transcript. In order to obtain a full length VP7-RV4 sequence, the cloning of VP7-RV4 was performed in order to eliminate failure function of the Taq polymerase (18).

Another drawback of T7 RNAP is that during the run-off, *in vitro* transcription has a tendency to create heterogeneity at the 5′ end of nascent nucleic acids with tandem guanosine residues. In our study, no tandem guanosine residue existed at the 5′ end. Additionally, T7 RNAP adds non-related nucleotides at the 3′ end of all transcripts, which result in the formation of many transcripts with heterogeneous 3′-terminus (19).

The proper solution for generating homogeneous RNA 3′ ends was to use hammerhead, hairpin or hepatitis delta virus (HDV) ribozyme upstream of the cleavage site. But the application of these methods has several limitations, including multiple steps of PCR to construct HDV ribozyme, and competition between the folding of the ribozyme HDV and the folding of the sequence of interest may lead to reduced cleavage efficiency. Further drawbacks are dephosphorylation of 2′, 3′ cyclic phosphate group at the 3′ end by T4 polynucleotide kinase, desalting and extraction of RNA (20).

Hence, we used restriction enzyme as an alternative to improve the RNA yield with correct 3′ terminus by cleavage of DNA at specific sequences, which subsequently reduce the amount of elongated or incomplete RNAs. Ease and accuracy of these extensions in cutting the expected sequence in the cloning process led to application of *Bstu-1* RE-site (GCGC) at 5′-end of the reverse primer in order to linearize the clone with blunt ends. In order to facilitate the enhancement of cleavage efficiency, two nucleotides (AT) were added upstream of the recognition site. (Reverse primer:

$$5\prime -\overset{\overset{\text{RE}\;\text{site}}{\text{B}\;\text{stu-}1}}{\underline{\text{AT}CGCG}}\text{GTCACATCGAACAATTCTAATCT}-3\prime ).$$

**Fig. 4:**
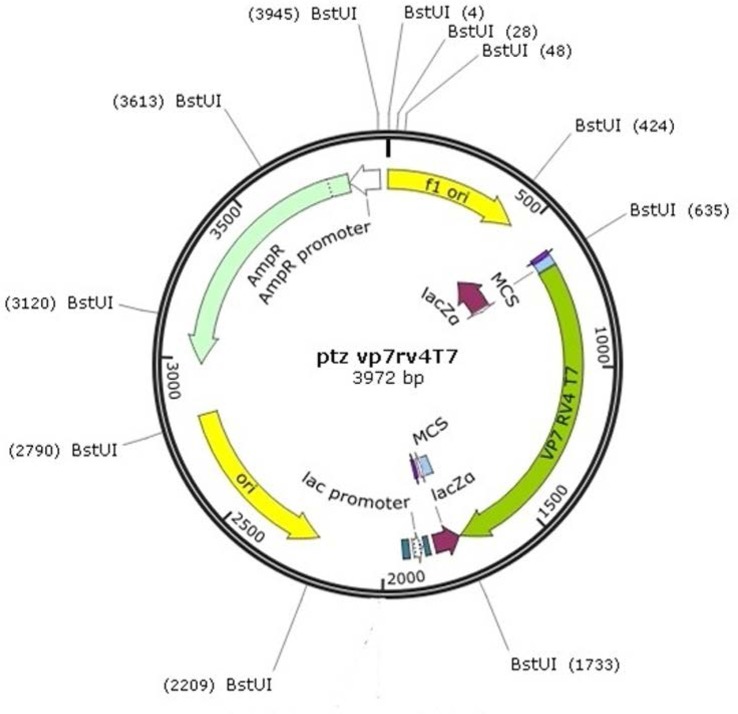
Map of PTZ 57 R/T-VP7 clone (designed with snap gene software): Sequence analysis indicates that the PTZ57 R/T-VP7 Clone had a full-length copy of segment 9 (VP7) of human rotavirus, and showed 99% homology with RV4 strain (GenBank Sequence ID: M64666.1). The *Bstu-1* restriction enzyme cleaves the PTZ57 R/T-VP7 Clone in the multiple positions. Two of these positions resulted in 635 and 1733 fragments that ultimately released the full-length of VP7 sequence from the plasmid

The ptz57/rt plasmid has multi-recognition sites for restriction enzyme *Bstu1*, but one of them existed exactly 14nt upstream of the 5′-end of the inserted DNA. With regard to the embedded CGCG sequence at 3′-end of gene of interest, it is clear that with one single digest enzyme, DNA-VP7 could easily be isolated (Fig. 4).

A consensus sequence, which conserves at 3′ end of the RV segments, must remain unchanged. The only problem persists after cutting with RE-*Bstu-1*, which is an additional guanine nucleotide remaining at position 1063 nt (the latest nucleotide of the VP7 sequence). First, it should be mentioned that the coding region sequence (CDS) of VP7 is from 49 to 1029 nt and this extra nucleotide is out of the translation region. Secondly, in the study of the ribozyme, better transcription efficiency was found when one extra nucleotide (A, T, C, and G) was placed before HDV ribozyme sequence (21, 22). In contrast, Schürer et al. (20) reported that by selecting any of the nucleotides in upstream HDV ribozyme, no significant efficient transcription will be observed. Therefore, it is unlikely that one guanine in the latest position of the sequence has a remarkable effect on the downstream application of the *in vitro* transcription, followed by a reverse genetic technique. Nonetheless, further investigations could help to ensure better evaluation of this hypothesis.

The quantity and quality of yielded RNA by *in vitro* transcription method, rely on some parameters, such as RNA template concentration, transcript size, time and temperature of the reaction. Based on these factors, the quality of synthesised RNA can be improved and adjusted by IVT (23). For pure DNA, A_260/280_ is broadly considered to be ∼1.8, but the ratio for pure RNA A_260/280_ is ∼2.0. In the present study, in order to generate an appropriate RNA yield with high purity (A_260/280_ was 2.02), the microtube containing reaction mixture was incubated at 37°C for 2 hours.

The protein blast (NCBI) alignment indicated that the expression of the VP7 was translated from the open reading frame 1 (ORF-1). The initial aa in the translated protein was Methionine-dependent and ended with Valine (arrows marked, Fig. 3). The aa sequence revealed a protein with 37.26 kDa and 98% identities with sequence ID: M64666.1. The results of aa alignment showed the presence of eight aa substitutions in the 326 aa of the VP7 protein. Two common factors including multi-infectious passages during cell culture and errors induced by Taq polymerase in RT-PCR reaction may result in the substitution of aa.

Some of the limitations of this examination cause an urgent need for superinfection or coinfection with a helper virus. Low survival of generated mRNA in the cell culture, requires appropriate selective antibodies system to confirm the validity and lack of powerful reporter system for tracking RNA-RV4 strain in cell culture. As well as, the transfection of IVT RNA-RV4 with a 5′ cap, significantly affects the viability of cells in the tissue culture. Also, the contamination of synthesised RNA with template DNA is inevitable, and no accurate procedure has been developed to clean up completely the excess of DNA from synthesised RNA. Lastly, Ribonucleases (RNases) are extremely steady, extra care should be taken while working with RNA.

In conclusion, the full-length sequence of VP7 ssRNA was developed using an *in vitro* transcription technique. Additionally, to establish an efficient approach and in order to obtain consensus ssRNA of VP7, the T7 promoter sequence was added at upstream and RE-site at downstream at 5′ and 3′ ends of the inserted DNA, respectively.

The current approach for ssRNA of VP7 by using *in vitro* transcription system promises a new design for production of resistant RV vaccine based on reverse genetic technology.

## References

[1] Manicone M Rende F Cavallari I Thoma-Kress AK Ciminale V. Expression of HTLV-1 Genes in T-Cells Using RNA Electroporation. Human T-Lymphotropic Viruses. Methods and Protocols. Methods Mol Bio 2017;1582:155–170. 28357669 10.1007/978-1-4939-6872-5_12

[2] Bonaldo MC Caufour PS Freire MS Galler R. The Yellow Fever 17D vaccine virus as a vector for the expression of foreign proteins: Development of new live flavivirus vaccines. Mem Inst Oswaldo Cruz 2000;95:215–223. 11142718 10.1590/s0074-02762000000700037

[3] Kobayashi T Ooms LS Ikizler M Chappell JD Dermody TS. NIH Public Access 2011;398:194–200. 10.1016/j.virol.2009.11.037 PMC2823833 20042210

[4] Taniguchi K Komoto S. Genetics and reverse genetics of rotavirus. Curr Opin Virol 2012 8 31;2(4):399–407. 22749758 10.1016/j.coviro.2012.06.001

[5] Khalili B Cuevas LE Reisi N Dove W Cunliffe NA Hart CA. Epidemiology of Rotavirus Diarrhoea in Iranian children. J Med Virol 2004;73:309–312. 15122809 10.1002/jmv.20092 PMC7166706

[6] Tate JE Burton AH Boschi-Pinto C Parashar UD Agocs M Serhan F Global, regional, and national estimates of rotavirus mortality in children <5 years of age, 2000–2013. Clin Infect Dis 2016;62(Suppl 2):S96–105. 27059362 10.1093/cid/civ1013 PMC11979873

[7] McDonald SM Nelson MI Turner PE Patton JT. Re-assortment in segmented RNA viruses: mechanisms and outcomes. Nature Reviews Microbiology 2016 1;14:448–460. 27211789 10.1038/nrmicro.2016.46 PMC5119462

[8] Ianiro G Delogu R Fiore L Ruggeri FM Pagani E dell'Alto Adige AS Genetic variability of VP7, VP4, VP6 and NSP4 genes of common human G1P[8] rotavirus strains circulating in Italy between 2010 and 2014. Virus Res 2016;220:117–128. 27130628 10.1016/j.virusres.2016.04.018

[9] Clarke E Desselberger U. Correlates of protection against human rotavirus disease and the factors influencing protection in low-income settings. Mucosal immunology 2015;8:1–17. 25465100 10.1038/mi.2014.114

[10] Sharma R Bora DP Chakraborty P Das S Barman NN. Circulation of group A rotaviruses among neonates of human, cow and pig: study from Assam, a north eastern state of India. Indian J Virol 2013;24:250–255. 24426283 10.1007/s13337-013-0153-0 PMC3784904

[11] Patton JT. Rotavirus diversity and evolution in the post-vaccine world. Discov Med 2012;13(68):85. 22284787 PMC3738915

[12] Liprandi F Gerder M Bastidas Z López JA Pujol FH Ludert JE A novel type of VP4 carried by a porcine rotavirus strain. Virology 2003;315:373–380. 14585340 10.1016/s0042-6822(03)00534-8

[13] Rahbarimanesh A Saberi H Modarres S Salamati P Akhtar-Khavari H Haghshenas Z Rotavirus VP4 Genotypes and Phylogenetic Analysis Among Strains Recovered From Children, Admitted With Acute Gastroenteritis in Bahrami Children's Hospital, Tehran, Iran. J Compr Ped 2014;5(1).

[14] Komoto S Taniguchi K. Genetic engineering of rotaviruses by reverse genetics. Microbiol Immunol 2013;57(7):479–486. 23692293 10.1111/1348-0421.12071

[15] Johne R Reetz J Kaufer BB Trojnar E. Generation of an Avian-Mammalian Rotavirus Reassortant by Using a Helper Virus-Dependent Reverse Genetics System. J Virol 2015;90:1439–1443. 26581988 10.1128/JVI.02730-15 PMC4719627

[16] Imburgio D Rong M Ma K McAllister WT. Studies of promoter recognition and start site selection by T7 RNA polymerase using a comprehensive collection of promoter variants. Biochemistry 2000;39(34):10419–10430. 10956032 10.1021/bi000365w

[17] Wichłacz A Łęgiewicz M Ciesiołka J. Generating in vitro transcripts with homogenous 3′ ends using trans-acting antigenomic delta ribozyme. Nucleic Acids Res 2004 2 1;32(3):e39. 14973333 10.1093/nar/gnh037 PMC373431

[18] Li Y Mitaxov V Waksman G. Structure-based design of Taq DNA polymerases with improved properties of dideoxynucleotide incorporation. Proc Natl Acad Sci USA 1999; 96:9491–9496. 10449720 10.1073/pnas.96.17.9491 PMC22236

[19] Salvail-Lacoste A Di Tomasso G Piette BL Legault P. Affinity purification of T7 RNA transcripts with homogeneous ends using ARiBo and CRISPR tags. RNA 2013;19:1003–14. 23657939 10.1261/rna.037432.112 PMC3683919

[20] Schürer H Lang K Schuster J Mörl M. A universal method to produce in vitro transcripts with homogeneous 3′ ends. Nucleic Acids Res 2002;30(12):e56. 12060694 10.1093/nar/gnf055 PMC117298

[21] Been MD. Cis-and trans-acting ribozymes from a human pathogen, hepatitis delta virus. Trends Biochem Sci 1994;19:251–256. 8073503 10.1016/0968-0004(94)90151-1

[22] Wu H nan Huang Z shun Mutagenesis analysis of the self-cleavage domain of hepatitis delta virus antigenomic RNA. Nucleic Acids Res 1992;20:5937–5941. 1461726 10.1093/nar/20.22.5937 PMC334457

[23] Gallo S Furler M Sigel RK. *In vitro* transcription and purification of RNAs of different size. CHIMIA 2005;59(11):812–816.

